# Dealing with Moral Challenges in Treatment of Transgender Children and Adolescents: Evaluating the Role of Moral Case Deliberation

**DOI:** 10.1007/s10508-020-01762-3

**Published:** 2020-06-26

**Authors:** Lieke Josephina Jeanne Johanna Vrouenraets, Laura A. Hartman, Irma M. Hein, Annelou L. C. de Vries, Martine C. de Vries, Bert A. C. Molewijk

**Affiliations:** 1 Curium-Department of Child and Adolescent Psychiatry, Leiden University Medical Center, Endegeesterstraatweg 27, 2342 AK Oegstgeest, The Netherlands; 2 Department of Medical Humanities, Amsterdam Public Health Research Institute, Amsterdam University Medical Centers, location VU University Medical Center, Amsterdam, The Netherlands; 3 Department of Child and Adolescent Psychiatry, Amsterdam University Medical Centers, location Academic Medical Center, Amsterdam, The Netherlands; 4 Department of Child and Adolescent Psychiatry, Amsterdam University Medical Centers, location VU University Medical Center, Amsterdam, The Netherlands; 5 Department of Pediatrics, Leiden University Medical Center, Leiden, The Netherlands

**Keywords:** Transgender, Moral case deliberation (MCD), Clinical ethics support (CES), Moral challenges, Gender dysphoria

## Abstract

Treatment teams providing affirmative medical transgender care to young people frequently face moral challenges arising from the care they provide. An adolescent’s capacity to consent, for example, could raise several issues and challenges. To deal with these challenges more effectively, several Dutch treatment teams started using a relatively well-established form of clinical ethics support (CES) called Moral Case Deliberation (MCD). MCD is a facilitator-led, collective moral inquiry based on a real case. This study’s purpose is to describe the teams’ perceived value and effectiveness of MCD. We conducted a mixed methods evaluation study using MCD session reports, individual interviews, focus groups, and MCD evaluation questionnaires. Our results show that Dutch transgender care providers rated MCD as highly valuable in situations where participants were confronted with moral challenges. The health care providers reported that MCD increased mutual understanding and open communication among team members and strengthened their ability to make decisions and take action when managing ethically difficult circumstances. However, the health care providers also expressed criticisms of MCD: some felt that the amount of time spent discussing individual cases was excessive, that MCD should lead to more practical and concrete results, and that MCD needed better integration and follow-up in the regular work process. We recommend future research on three matters: studying how MCD contributes to the quality of care, involvement of transgender people themselves in MCD, and integration of CES into daily work processes.

## Introduction

Transgender care is being offered to an increasing number of children and adolescents (Aitken et al., 2015; Chen, Fuqua, & Eugster, 2016; de Vries & Cohen-Kettenis, 2012; Wood et al., 2013). The incongruence they experience between assigned sex and identified gender is called gender dysphoria (GD) and may be diagnosed according to the DSM-5 when accompanied by distress (American Psychiatric Association, 2013). Children and adolescents with GD are usually treated by a team consisting of child and adolescent psychiatrists and psychologists, (pediatric) endocrinologists, gynecologists (for fertility advice), and surgeons (for gender affirmative surgery). In this article, the term “adolescent” refers to children and adolescents in whom puberty has started. The start of puberty is defined as the appearance of Tanner stage 2–3 in boys (G2–3) and Tanner stage 2 in girls (M2). This article uses the term “transgender adults/adolescents/children” to refer to persons diagnosed with GD.

Transgender teams base their treatment decisions on internationally recognized clinical guidelines set by professional transgender care associations. In practice, these guidelines are often adapted to local situations (Coleman et al., 2012; Hembree et al., 2017). A Dutch treatment protocol for transgender adolescents, sometimes referred to as “the Dutch model,” was the first in the world to explicitly describe medical transgender treatment of young adolescents (Cohen-Kettenis, Steensma, & de Vries, 2011). In the Dutch model, the eligibility criteria for such treatment are: a long history of GD, no psychosocial problems interfering with assessment or treatment, sufficient family or other social support, and the appearance of Tanner stages 2-3 indicating the onset of puberty (de Vries & Cohen-Kettenis, 2012; Delemarre-van de Waal & Cohen-Kettenis, 2006; Kreukels & Cohen-Kettenis, 2011; Shumer & Spack, 2015). Over the years, these eligibility criteria have not changed and are also part of the Standards of Care and Endocrine guidelines (Coleman et al., 2012; Hembree et al., 2017). Also, despite an enormous increase, main characteristics (with the exception of a shift in sex ratio with an overrepresentation of assigned females) of the referrals did not change over the years (Arnoldussen et al., 2019). Mental health is an inseparable part of the clinical care of adolescents in the Netherlands. Besides the possible medical treatment, the clinical care requires an ongoing relationship with a psychologist and/or psychiatrist from the team. In many cases, it will also involve a local mental health specialist. In this article, we use the term “gender affirmative treatment” which includes the medical gender affirmative part which is always preceded by assessment, and it is always accompanied by mental health counseling. This counseling consists of regular sessions in which information and advice is provided, and psychological and/or family support is given depending on the individual needs.

Professionals at transgender clinics are frequently confronted with controversies and moral challenges arising from the care they provide for young people (Drescher & Pula, 2014; Vrouenraets, Fredriks, Hannema, Cohen-Kettenis, & de Vries, 2015). In general, moral challenges arise when professionals doubt as to the morally right course of action to take (Molewijk, Hem, & Pedersen, 2015). One type of moral challenge is a moral dilemma. In moral dilemmas, there are two mutually exclusive moral imperatives, neither of which is unambiguously desirable or acceptable (Stolper, Molewijk, & Widdershoven, 2016). Many clinical dilemmas fall into this category because they have a moral dimension. The moral dilemmas often faced by treatment teams working with transgender children and adolescents (and adults) include: (1) What should the professional do if the he/she is in doubt whether the adolescent fully comprehend the implications of gender affirmative treatment?; (2) When is a psychiatric disorder so serious that we should not start gender affirmative treatment?; and (3) Must we reach a multidisciplinary team consensus about the whole treatment before treatment commences, or is it justifiable for discipline X to start part Y of the whole gender affirmative treatment before a consensus has been reached? (Byne et al., 2012; Gerritse et al., 2018; Milrod, 2014; Stein, 2012; Vrouenraets et al., 2015).

Several reasons exist on why transgender care for adolescents entails a particularly large number of moral challenges. To begin with, transgender care for young people is a relatively new domain, on which there are many different normative views. These exist at both a professional and societal level (Byne et al., 2012). In addition, the normative views on the treatment of child and adolescent GD are continuously evolving (Byne et al., 2012). Another common source of moral challenges in transgender care is the multidisciplinary nature of such care and the resulting divergence of professional views on the appropriate treatment criteria. Also, many of the long-term effects of administering medications or refraining from puberty suppression are as yet unknown, causing treatment uncertainty (Stein, 2012; Vrouenraets et al., 2015). Furthermore, medical treatment of transgender adolescents is seen as an intervention in a physically healthy and, in most cases, still developing body. Lastly, the adolescents undergoing such treatment are considered to be not yet fully developed in a psychological and cognitive sense (Byne et al., 2012; Crone, 2016; Moshman, 2017). These factors raise doubts about the potential risks of suppressing pubertal development in terms of physical development, brain growth, and the building of a consistent gender identity (Cohen-Kettenis, Delemarre-van de Waal, & Gooren, 2008).

In many clinical settings, health care providers are assisted in dealing with moral challenges and questions through structural clinical ethics support (CES) (Schildmann, Gordon, & Vollmann, 2010). Various CES methods are available, including individual consultations with an ethicist and ethics committee meetings. None of the current CES methods are versatile enough to cover the entire range of challenges and questions debated in the clinical context (Steinkamp & Gordijn, 2003). In the Netherlands, a relatively well-established type of CES is Moral Case Deliberation (MCD) (Dauwerse, Weidema, Abma, Molewijk, & Widdershoven, 2014; Molewijk, Abma, Stolper, & Widdershoven, 2008a). MCD is a facilitator-led, collective moral inquiry by health care providers that focuses on a concrete moral question connected to a real clinical case (Dauwerse et al., 2014; Stolper et al., 2016). The aim of MCD is to create a dialogue that enables the treatment team to pursue a critical, yet constructive moral inquiry into the moral challenge at hand. The MCD facilitator uses a specific conversational method to structure multidisciplinary team meetings in which participants critically reflect on both current and past cases. Examples of such conversation methods are the Dilemma Method or the Socratic Dialogue (Molewijk et al., 2008a). The MCD method is designed to encourage health care providers to consider different viewpoints on the concrete moral challenges they experience in their everyday clinical work. MCD can stimulate reflection and deepen decision-making processes. It is not meant to substitute any aspect of the regular care or decision-making process, but rather as a supplement to these processes. MCD does not have any decision-making mandate and does not replace any decision-making mandate. However, various evaluative studies indicate that MCD can help improve a team’s handling of moral challenges, increase the moral competency of health care providers, strengthen multidisciplinary cooperation, and facilitate the development, adjustment, and implementation of guidelines and policies (Hem, Pedersen, Norvoll, & Molewijk, 2015; Janssens, van Zadelhoff, van Loo, Widdershoven, & Molewijk, 2014; Molewijk, Verkerk, Milius, & Widdershoven, 2008c; Weidema, Molewijk, Kamsteeg, & Widdershoven, 2015). In MCD sessions, a certified MCD facilitator supports a joint reasoning process, fostering a systematic and critical yet constructive dialogue while keeping the group’s focus on the moral dimension of the case without giving advice (Stolper, Molewijk, & Widdershoven, 2015). Among the facts, the following can be included in the dialogue: protocols, existing policy and guidelines, legal regulations, and professionals’ own practical experiences, and normative considerations. Preferably, an MCD session should take a multidisciplinary approach because this brings to light different viewpoints on the moral issue at hand. Conclusions and insights gained from MCD sessions may be used to develop or adjust multidisciplinary treatment policies in the future.

The obvious moral dimension of transgender care for adolescents creates a niche for specific CES services. Yet, it is not yet known which CES methods are most suitable for the particular moral dilemmas that arise in transgender care for young people. Vrouenraets et al. (2015) conducted an interview study in which clinicians reported a need to structurally discuss moral challenges among their multidisciplinary transgender teams. This led to the initiative of using MCD as one of the CES methods for dealing with moral challenges in transgender care in the Netherlands.

The aim of this study is to describe how Dutch transgender care providers evaluated the usefulness of MCD in dealing with moral challenges in the multidisciplinary transgender clinical treatment of adolescents. For this purpose, we have conducted a mixed methods evaluation study to answer the following questions:

1. How valuable do transgender care professionals evaluate MCD as CES, or in other words, what is their opinion of MCD?
2. What kind of change do MCD participants perceive after MCD sessions?
3. What recommendations can the interviewed professionals offer with respect to the future use of MCD?
4. Which MCD outcomes do the professionals hope to see when taking part in MCD sessions, and which MCD-related outcomes do they actually experience during MCD sessions and afterward in their daily work?

## Method

### Participants and Procedure

During the period when the data for this study were collected, from February 1, 2014, until April 13, 2015, there were two transgender clinics offering gender affirming medical treatment in the Netherlands. These were (1) the Center of Expertise for Gender Dysphoria at the Amsterdam University Medical Centers, location VU University Medical Center (VUmc) in Amsterdam, which offered care for children, adolescents, and adults; and (2) the transgender clinic at Curium-Leiden University Medical Center in Leiden, which offered care for children and adolescents. At both locations, specialists in child and adolescent psychiatry and psychology, endocrinology, and pediatric endocrinology worked in multidisciplinary teams. The two teams followed the same diagnostic and treatment procedures, had similar protocols, and regularly held joint meetings. In 2013, the board overseeing transgender clinical care in the Netherlands introduced MCD to complement the two teams’ regular care and decision-making processes. The aim was to create opportunities for the clinicians to thoroughly reflect on the moral dilemmas they faced in difficult cases. Therefore, MCD was only intermittently used by the transgender teams.

In the period from October 2013 until January 2015, the two teams convened for nine joint meetings during which a total of 17 MCD sessions were held. During six of these joint meetings, two or three parallel MCD sessions were held, depending on the number of participants. In each of these parallel MCD sessions, a different case was discussed. We analyzed six of these MCD sessions for this study. The sessions were led by trained and certified MCD facilitators employed by the Department of Medical Humanities of the Amsterdam University Medical Centers, location VUmc, which is responsible for all CES services at that institution. The sessions analyzed in this study used the MCD dilemma method, which consists of the 10 steps listed in “Appendix 1” of this study (for a detailed example of an MCD dilemma method see the paper of Stolper et al., 2016). Furthermore, health care providers from both teams completed a validated questionnaire on perceived MCD outcomes (Euro-MCD; Svantesson et al., 2014) and participated in individual interviews and focus groups.

The participants in this study were members of the Amsterdam and Leiden transgender teams. Both teams included endocrinologists and specialists in child and adolescent psychiatry and psychology. The Amsterdam team also included surgeons and gynecologists. The transgender clinic in Amsterdam had approximately 30 team members when this study was conducted. The team in Leiden had seven members in the same period. Ten to fifteen team members participated in each MCD session. We individually interviewed three specifically selected team members from each team. The interviewees were two child and adolescent psychiatrists, two child and adolescent psychologists, and two endocrinologists. The interviewees had different levels of experience with transgender youth care. Therefore, the six interviews can be considered representative of the larger group of participants across the two teams in terms of their range of disciplines and experience level.

In addition to the interviews, we conducted two focus groups: one with nine members of the Amsterdam team, and one with six members of the Leiden team. Each focus group consisted of a representative part of the multidisciplinary team from which it was derived. In order to make the groups representative, we included members with different disciplines and levels of experience in the field of transgender child and adolescent care. No more information about the participants can be provided here because this could compromise the anonymity of some participants, since certain functions are performed by only one or two members of a given team. The focus groups were used to refine and validate the findings from the interviews. As part of the mixed method design, we conducted a small cross-sectional survey using the Euro-MCD questionnaire at two joint meetings of the Amsterdam and Leiden teams (T0 and T1). Twenty-eight professionals from the Amsterdam team and six from Leiden completed the Euro-MCD questionnaire at T0 (*n* = 34). Twenty professionals on the Amsterdam team and two from Leiden completed the questionnaire at T1 (*n* = 22). To protect the privacy of the respondents, their names were not collected. Therefore, it was not possible to match those who participated in this survey twice and no longitudinal data were collected (Table 1).

**Table 1:** Overview number of participants

Team	Team members (N)	Participants (N)				
		In each MCD session	Individual interviews	Focus groups	Euro-MCD questionnaire at T0	Euro-MCD questionnaire at T1
Amsterdam	30	*	3	9	28	20
Leiden	7	*	3	6	6	2
Total	37	10 to 15	6	15	34	22

*No data available

### Design

We used a mixed method research design consisting of a qualitative and a quantitative component. In each of these, the stakeholders evaluated the MCD sessions (Morse, Niehaus, Wolfe, & Wilkins, 2006). We collected data in four different ways (see Table 2). To answer the first, second, and third research questions, we used the qualitative data recorded on audiotapes of the MCD sessions and collected during the individual interviews and focus groups. In order to answer the fourth research question, we used data obtained from the Euro-MCD questionnaires, the individual interviews, and the focus groups.

**Table 2:** Overview of the dataset

Data collection method	Moment in time	*N*	Type of data	Amount
Audiotapes of MCD sessions		6	Transcript	4
			Summary to verify accuracy	6
Individual interviews		6	Transcript	6
			Summary to verify accuracy	6
			Observational note	4
Focus groups		2	Transcript	2
			Summary to verify accuracy	2
			Observational note	2
Euro-MCD questionnaire	T0; prior to the MCD sessions	34	Questionnaire	34
	T1; after participating in two to four MCD sessions	22	Questionnaire	22

#### Audiotapes of MCD Sessions

From October 2013 until January 2015, the transgender teams for children and adolescents organized six MCD sessions which were incorporated into this research project. The MCD sessions were structured according to the dilemma method (Stolper et al., 2016), lasted from 50 to 116 min and were audiotaped. We transcribed verbatim four MCD sessions. The MCD facilitators wrote a summary of all six MCD sessions and sent this back to the MCD participants to verify its accuracy.

#### MCD Evaluation in Individual Interviews

We conducted semi-structured individual interviews with six members of both the Amsterdam and Leiden transgender teams in February 2015. We formulated our initial interview topics after reviewing the relevant literature and then examining how the teams had experienced the MCD sessions (Janssens et al., 2014). The interviews contained general topics and no closed-ended questions. In the interviews, we asked the participants to elaborate on their personal experiences with MCD and the method’s effects, or lack thereof, at the patient level or protocol level.

One of the researchers conducted the interviews, and a participating intern took notes during the four interviews. The interviews lasted from 60 to 90 min and were audiotaped and transcribed verbatim. A summary of each individual interview was sent to the participant to verify its accuracy.

#### Evaluation of MCD by Focus Groups

We conducted two focus groups in March 2015. During these focus groups, we presented several anonymous quotes from the individual interviews to trigger the conversation. We asked the group participants whether they could identify and/or agree with the quotes and invited the participants to express any other views they held on the use of MCD. Our goal was to establish a dialogue among the team members rather than maintaining a strict question-and-answer format.

Two of the researchers facilitated each focus group. During one focus group, a second researcher made observatory notes, while in the other focus group the observatory notes were taken by a participating intern. The focus groups lasted from 100 to 120 min and were audiotaped and transcribed verbatim. A summary of each focus group discussion was sent to the transgender team to verify their accuracy.

#### MCD Evaluation by Means of the Euro-MCD Questionnaire

The Euro-MCD questionnaire is a qualitatively validated questionnaire (Svantesson et al., 2014). This instrument was developed to measure: (1) the perceived importance of MCD outcomes prior to MCD sessions (T0); (2) the experienced MCD outcomes during the MCD sessions (T1); and (3) the experienced MCD outcomes within daily work after a series of two to four MCD sessions (T1). The 26 outcomes cover six domains: enhanced emotional support; enhanced collaboration; improved moral reflexivity; improved moral attitude; improvement at organizational level; concrete results concerning the care or treatment (see “Appendix 2” for an example). Participants were asked at T0 how important they found these 26 outcomes (on a 4-point Likert scale: very important; important; somewhat important; not important); and at T1 whether they experienced these outcomes (on a 4-point Likert scale: experienced to a great extent; experienced to a reasonable extent; experienced to some extent; did not experience). The Euro-MCD questionnaire also contains some open-ended questions asking, for instance, what outcome the respondent expected (without reading the 26 outcomes) and how the respondent prioritized the five most important outcomes. Finally, the Euro-MCD questionnaire also collects some of the participants’ general characteristics (e.g., age, sex, specialty, institution). We obtained these empirical data between April 2014 and October 2014, during two joint meetings of the Amsterdam and Leiden teams.

### Data Analysis

#### MCD Sessions, Individual Interviews, and Focus Groups

Following an initial open reading of the qualitative data, the authors independently identified some preliminary sub-themes. Then, through a deliberative process, the authors redefined the initial sub-themes into main themes until they reached a consensus. This hermeneutic analysis (Miles & Huberman, 1994; Stake, 2005), partly inspired by the interview guide, resulted in four main themes: (1) Positive experiences with MCD; (2) Critical remarks about MCD; (3) Changes in daily work processes after MCD; (4) Recommendations for the future use of MCD or MCD elements in daily work processes.

Two authors conducted an additional round of analyses to assess whether the themes or sub-themes enabled them to accurately subdivide the outcome of qualitative data. Following this assessment, they kept the four initially defined main themes unchanged. We then re-analyzed the transcripts of the MCD sessions, the focus groups, and the individual interviews and selected representative quotations for each of the defined themes, taking equal care to draw quotations from all data sources.

#### Integration of MCD Sessions, Individual Interviews, Focus Groups, and Euro-MCD Questionnaires

In the present study, the results obtained from each of the four methods to produce four sets of findings were collected and analyzed separately. Subsequently, these data were combined in a process called “triangulation” (O’Cathain, Murphy, & Nicholl, 2010). Combining the data of all four methods consisted of comparing the qualitative and quantitative data and reflecting upon the similarities and differences found.

### Research Ethics

Prior to the individual interviews, focus groups, and MCD sessions, we gave all participants oral instructions to inform them of the voluntary nature of their participation; they were told that they could withdraw from the interview, focus group or MCD session at any time with no explanation required. Furthermore, we emphasized that the data we were about to collect would remain anonymous. Upon each interview, focus group, and MCD session, we obtained the participants’ oral informed consent for their participation and tape recording. No clinician refused to take part in an interview or MCD sessions. Due to the small size of the teams, we cannot provide more details about the participants in this paper without potentially compromising our commitment to confidentiality.

## Results

Below, we describe the data obtained from MCD sessions, individual interviews, focus groups, and Euro-MCD questionnaires. We largely answered the first three research questions based on the qualitative data, and the last research question based on the quantitative data.

### How Valuable Do Transgender Care Professionals Evaluate MDC as CES, or in Other Words, What Is Their Opinion of MCD?

First, we will present the positive experiences team members reported having with MCD. This will be followed by an outline of their critical remarks about MCD.

#### Positive experiences with MCD

*Becoming aware of others’ perspectives and interests*

Nearly all interviewed participants reported that they had gained more insight into the perspectives and interests of the other stakeholders in the case discussed. Some found it valuable to hear how professionals from other disciplines viewed the case.

You always look at a situation from a certain perspective. Since people from different disciplines take part in Moral Case Deliberation sessions, you learn to view a situation from another perspective (Individual interview).

Sometimes patients and health care providers look at a certain situation in a similar way, but there are also times when they view things differently. Moral Case Deliberation brings that to the surface (Individual interview).

#### Dialogue Among All Instead of Discussion Among Some

Participants often voiced appreciation of the fact that all MCD participants were encouraged to contribute and that disagreements were discussed in a less polarizing way than is customary. Due to the structure of the dilemma method and the MCD facilitator’s role, participants have to take the time to listen to others instead of attempting to convince others of their own point of view. This enabled the participants to structure the relevant arguments and take a more open attitude toward other perspectives. The dilemma method encourages the participants to incorporate the arguments and merits of “the other side” of the dilemma rather than to try to discredit a colleague’s opinion.

Sometimes our disagreements are expressed with so much emotion, that you can get the impression that the one [team member] who expresses the strongest emotion has the truth on their side. Moral Case Deliberation is good at neutralizing the emotions and returning participants to the crux of the matter. Moral Case Deliberation encourages you to acknowledge the merits of both sides of a dilemma (Individual interview).

I like the fact that you do not have to persuade anyone else of your point of view [in a Moral Case Deliberation session], and that it is possible for several opinions to coexist. This doesn't happen in regular meetings, because in those meetings decisions have to be made and all you want is to get your own point of view across (Focus group).

#### Paying Closer Attention to Your Own Arguments and Contextual Factors, Rather than Blindly Following Protocol

Another frequently mentioned positive experience with MCD was that it enabled the teams to explore in great detail how they had handled a given protocol and why they had done so, which gave them deeper insight. Furthermore, the teams often used MCD sessions to discuss cases or requests not covered by the existing protocol. During the MCD sessions, the participants exchanged ideas on the question, “Which rules do we view as absolute and which can be handled with greater flexibility, and when it comes to the latter, under what conditions may we do so?”

Certain decisions can be found in our protocol, but in Moral Case Deliberation, you are encouraged to ask questions like: “Why do we do things that way?” and “What, precisely, is the risk if we take this step?” (Individual interview)

In the collaboration between Amsterdam and Leiden, we also look at the boundaries of the protocol and how we handle these boundaries in practice (Individual interview).

#### Extra Time for Reflection Is Important and Useful

The participants appreciated the time MCD offers for talking about problematic cases. They said this was particularly important because of the current cultural emphasis on efficiency; in general meetings, for instance, less time is available for exchanging viewpoints or discussing difficult cases.

Nowadays, with all the pressure to work as efficiently as possible, it is becoming even more important to set aside moments to discuss a difficult situation (Individual interview).

After all, transgender care is a subject surrounded by many controversies and one in which a lot of pressure is applied by parents, children, and interest groups. The topic is sometimes so fraught that it is helpful to put on the brakes now and then so we can discuss matters calmly first (Focus group).

One participant reported having gotten a fresh perspective thanks to MCD. This individual remarked that professionals with more experience tend to work faster, but by applying previous experience to new situations, they run the risk of being blind to potentially different characteristics in these new situations. MCD sessions helped restore this participant’s sensitivity to the particularity of the case at hand.

Over time, you sort of put blinkers on, because similar cases appear and you do not have the time to really consider the case because of your busy schedule. You do not have time to think. That really is a problem (Individual interview).

### Critical Remarks About MCD

#### The Tempo Is Sometimes Too Slow and the Sessions Last Too Long

In MCD sessions, every participant is actively encouraged to contribute. A couple of participants characterized themselves as goal-oriented and said they were frustrated by the length of the deliberations, especially when they felt that participants were repeating a certain point that had already been discussed.

Sometimes I get the feeling that we know what our goal is, and we could get there faster. Maybe it is necessary to involve the whole group in a deliberation, but sometimes I find it frustrating that it takes so long (Individual interview).

#### Discussing Several Similar Cases in Succession Gives Less Insight

MCD requires an organization to invest a certain amount of time, so some participants questioned whether MCD was worth the time investment. Some participants said they considered the nature of the cases themselves to be an important criterion to determine whether MCD was worth the time it takes. Some noted that team members felt a certain “fatigue,” especially when the case under discussion was not recognizable to everyone or when it had the same characteristics as a previously discussed case.

Here we go again, another patient who has characteristic X (Individual interview).

Others who had not experienced this themselves could imagine it might happen to them in the future.

You cannot organize a Moral Case Deliberation for every patient, so you need to constantly weigh whether it is a worthwhile time investment. So far, we have only discussed cases that offered us new insights, but I can imagine that sooner or later we will reach a saturation point (Individual interview).

These different experiences show that what participants see as “the same” or “similar” was often ambiguous and unclear.

#### Insufficient Follow-up

Some participants mentioned that there was insufficient follow-up after MCD. The MCD sessions frequently produced new insights and signals, but it was unclear what follow-up actions were being taken if any and who should be responsible for following up. For instance, one difficult request which had long been expressed was discussed in an MCD session, but subsequently kept coming up in regular team meetings and yet no action was taken.

And more in general, when we do reach some kind of conclusion, there’s often no concrete follow-up. And that’s a shame (Individual interview).

This caused some irritation. We talked about this case in a Moral Case Deliberation, so we knew we should see some kind of an outcome, but we still did not know what the outcome would be (Individual interview).

#### Perhaps Taking Time to Deliberate Is All that Really Counts, and Not this Particular Method?

Most participants said taking more time to deliberate a case was worthwhile and that it helped them deal with moral challenges in their work. Some asserted that it was not the particular method (in this case, MCD) that had made the difference, but merely the fact that the team was taking ample time to deliberate a case.

I wonder whether this [giving more thought to a case and asking more constructive questions regarding the case] is due to the [method] Moral Case Deliberation or due to the fact that we have taken time for each case. The fact that we talked about one patient for an hour and a half is in itself enormously valuable. That is independent of the method used. Normally, we would never do that (Focus group).

### What Kind of Change Do MCD Participants Perceive After MCD Sessions?

#### Changes in Treatment Decisions

Some participants reported that they changed treatment plans because of a discussion in an MCD session. After the MCD session in question, some team members who were treating a particular patient held a small meeting about the MCD’s results and its implications for the individual treatment plan. They subsequently called the patient in and elaborated on their new treatment plan.

After the Moral Case Deliberation session, we made another decision (…) a different policy was applied (…) instead of prescribing puberty suppression treatment [in accordance with the adolescent protocol] to an adolescent aged 17.5 years, we decided to not prescribe any medical treatment at that moment. But instead, we let the adolescent start with cross-sex hormones when he turned 18, following the adult protocol (…) we are talking about a concrete change, with concrete conditions. Furthermore, a concrete policy has emerged from it. Different from what our local protocol suggests and different from what was discussed earlier with the patient (Individual interview).

#### Learning Effects in MCD Sessions and Outside

Participants attributed several learning effects to MCD.

The teams grew more experienced with the specific method which helped them get to the heart of a case sooner.

The repetition of the dilemma method enables you to get to the heart of the case and its policy more quickly. We are internalizing the method more and more, now that we have used it several times (Focus group).

Thorough analysis of a particular case helped team members deal with similar cases. Some participants reported having become more aware of certain issues after they were discussed in an MCD session. They reported reacting more adequately to similar situations thanks to the discussion they had held in MCD.

Within [a] Moral Case Deliberation [session], you take the content and that particular patient into account. What are the criteria and arguments on which we base a decision? And what are the advantages and disadvantages? This also has a learning effect for cases that are similar (Individual interview).

I notice that you internalize what has been talked about [during a Moral Case Deliberation session] and that you bear that in mind during subsequent contact with patients (…) In my opinion, that is an important added value of a Moral Case Deliberation session: that you learn to break through the standard pathways and ideas (Focus group).

- Participants became aware of the normative dimension of a problem.

Due to Moral Case Deliberation, I realized that our care is full of moral dilemmas. I first had the impression that it was all more or less determined. This realization is sometimes unsettling, but also a good thing (Individual interview).

#### Improved Decision-Making Process

Most participants stated that the quality of treatment decisions improved in different ways.

You’re forced to think about it [the case at hand] systematically and not to make decisions too quickly. It helps you to carefully consider things, and this improves the quality of the decision (Individual interview).

The MCD method structured the conversation. All pro and con arguments are given a place, creating coherence. The MCD framework contributed to more nuanced judgments in which the separate and sometimes conflicting values and norms are considered and weighed.

Some participants said they had learned to give a better explanation and justification for certain decisions. They reported being able to explain more precisely why they had made a certain decision and had learned to give substantiated arguments to the patient, family, and colleagues.

The decisions we make are very impactful (…) you do have the responsibility to the patient to explain why you are starting treatment, or why not. (…) [Therefore] it is important to make the underlying idea explicit. (… So I think that, especially in this population, it is good to have some sort of mini Moral Case Deliberation on very difficult cases (Individual interview).

### What Recommendations Can the Interviewed Professionals Offer With Respect to the Future Use of MCD?

#### Structurally Embedding MCD Sessions

Most participants recommended that MCD sessions be structurally embedded into regular interdisciplinary meetings. Most expressed a belief that structurally embedding MCD sessions would increase the successful use of MCD. Furthermore, some participants said they would appreciate the opportunity to hold ad hoc MCD sessions on urgent cases in their regular clinical work. However, others argued that it would be too difficult in practical terms to schedule such an ad hoc MCD session.

I wonder whether there will be a Moral Case Deliberation session if you do not structurally embed it in your organization. Especially when it is with a large group, it is difficult to find a date for an ad hoc Moral Case Deliberation session. I think that Moral Case Deliberation is therefore more likely to succeed if we do it structurally, in regular interdisciplinary meetings (Individual interview).

#### Training Team Members as MCD Session Facilitators

Some participants said it would be of great value to have a colleague trained as an MCD session facilitator because this would make it easier to organize ad hoc MCD meetings. Others doubted whether this colleague could show the neutrality required of an MCD facilitator, because he or she would also be a member of the treatment team.

It would be good to have the expertise in-house. That would lower the threshold for an ad hoc Moral Case Deliberation session (Focus group).

I wonder whether a member of the team can achieve the neutrality needed to be a facilitator of Moral Case Deliberation sessions (Focus group).

#### Using Elements of MCD in Other Meetings

Some participants saw the potential value of using elements of MCD to structure discussions in other meetings. The participants mentioned various steps of the dilemma method that they wanted to introduce as part of regular team meetings (such as brainstorming on alternatives and the table with norms and values for the various treatment or decision options).

One of the great things I find are the questions: “What are your alternatives?” [and] “Are there alternatives?” These are questions that could also be considered during regular meetings. What is the broad range of available options? Our discussions are often just yes or no (Individual interview).

#### Better Follow-up

The interviews and focus groups led to some concrete recommendations for improving the use of MCD in the daily work of transgender teams. These recommendations were: to better monitor the to-do lists and other conclusions agreed upon in MCD sessions; to better coordinate team members’ tasks and responsibilities after these sessions; to discuss the results and actions agreed upon in MCD sessions during regular treatment team meetings and to ensure that current guidelines and policies reflect these results.

After conducting a Moral Case Deliberation session, how can we ensure that we get a fixed moment to reflect on the question: “How do we proceed with what has been discussed in relation to our final decision making?” Maybe we could do that during a moment of feedback to the team (Individual interview).

### Which MCD Outcomes Do the Professionals Hope to See When Taking Part in MCD Sessions, and Which MCD-related Outcomes Do They Actually Experience During MCD Sessions and Afterwards in Their Daily Work?

Prior to starting their MCD sessions, 34 team members completed the Euro-MCD questionnaire. This survey asked these professionals what outcomes they expected MCD to have, and how important these specific outcomes were to them. The questionnaire was based on 26 pre-defined MCD-related outcomes (T0) (Svantesson et al., 2014). After the team members had attended two to four MCD sessions, they were asked once again to complete the Euro-MCD questionnaire (T1), but this time regarding, (1) which MCD-related outcomes they had actually observed during the MCD sessions overall; and (2) which MCD-related outcomes they had observed in their daily work after the MCD sessions (*n* = 22).

Table 3 provides an overview of the most important results of the Euro-MCD questionnaire at T0 and T1, by means of the outcomes as described in the fixed-choice questions.

**Table 3:** Outcomes of Euro-MCD questionnaire at T0 and T1; outcomes as described in the fixed-choice questions of Euro-MCD questionnaire (including the corresponding number)

Outcomes described most often as “important” or “very important” before starting MCD sessions (T0, *n* = 34)	Percentage assessed as “important” or “very important”
8. Better mutual understanding of reasoning and behavior	97%
2. More open communication among co-workers	91%
13. Enables me and my co-workers to decide on concrete steps to manage ethically difficult situations	89%
Outcomes described most often as “not important” or “a little important” before starting MCD sessions (T0, *n* = 34)	Percentage assessed as “not important” or “somewhat important”
12. Enhances my understanding of ethical theories (ethical principles, values and norms)	66%
4. Enables me to better manage the stress caused by ethically difficult situations	63%
17. I listen more seriously to others’ opinions	61%
Most frequently experienced outcomes during the MCD sessions (T1, *n* = 22)	Percentage assessed as “experienced to a reasonable extent” or “experienced to a great extent”
10. My co-workers and I become more aware of recurring, ethically difficult situations	85%
8. Better mutual understanding of reasoning and behavior	84%
2. More open communication among co-workers	82%
Least often experienced outcomes during the MCD sessions (T1, *n* = 22)	Percentage assessed as “not experienced” or “experienced to some extent”
19. Boosts my self-confidence when managing ethically difficult situations	67%
12. Enhances my understanding of ethical theories (ethical principles, values and norms)	65%
4. Enables me to better manage the stress caused by ethically difficult situations	63%
Most frequently experienced outcomes in daily work after the MCD sessions (T1, *n* = 22)	Percentage assessed as “experienced to a reasonable extent” or “experienced to a great extent”
9. I see ethically difficult situations from different perspectives	55%
10. My co-workers and I become more aware of recurring, ethically difficult situations	55%
24. Enhances mutual respect among co-workers	55%
Least often experienced outcomes in daily work after the MCD sessions (T1, *n* = 22)	Percentage assessed as “not experienced” or “experienced to some extent”
4. Enables me to better manage the stress caused by ethically difficult situations	85%
13. Enables me and my co-workers to decide on concrete steps to manage ethically difficult situations	75%
12. Enhances my understanding of ethical theories (ethical principles, values and norms)	75%

When completing the questionnaire at T0, prior to participating in the MCD sessions, almost all respondents rated improvement in the areas of “open communication among co-workers” and “mutual understanding of reasoning and behavior” as “important” or “very important.” When they completed the questionnaire at T1, most participants reported having experienced more open communication and mutual understanding during the MCD sessions. Furthermore, most team members experienced an improvement in “becoming aware of recurring, ethically difficult situations” both during the MCD sessions and in their daily work after these sessions.

The results from T0 showed that respondents rated the MCD-related outcomes “to enhance my own understanding of ethical theories” and “to manage stress related to the cases better” as “not important” or “somewhat important.” In line with these results, the respondents reported at T1 that they had either not experienced these two outcomes, or only experienced them to some extent, during the MCD sessions and afterward in their daily work.

A large majority of respondents initially rated an improvement in “enabling team members to decide on concrete actions in order to manage ethically difficult situations” as “important” or “very important.” At T1, however, most respondents reported that they had not experienced this outcome in their daily work subsequent to the MCD sessions, or only to some extent.

Most participants experienced an increase in mutual respect among team members in their daily work after the sessions. During the individual interviews and focus groups, several participants mentioned that participating in an MCD session enhanced mutual trust on the team. They attributed this to having learned more about the thinking behind their colleagues’ actions at work because the MCD sessions encouraged individuals to express their own opinions rather than team opinions. Furthermore, the results of the Euro-MCD questionnaire revealed that the majority of care providers experienced an “enhancement in mutual respect among the team members” after participating in the MCD sessions, and that they “became more aware of the stakeholders’ different perspectives” and of “recurring, ethically difficult situations.”

I think that Moral Case Deliberation is a very good way of bringing about greater mutual trust. You usually get closer (…) during Moral Case Deliberation sessions there are no team opinions, but all individual opinions–that strengthens your sense of connection (Focus group).

Some participants stated that mutual trust is especially crucial in transgender care because several treatment steps are deeply interwoven, requiring close cooperation between the various disciplines involved in the transgender care trajectory.

The amount of cooperation [between different disciplines] in transgender care is uncommon in the medical world. A doctor normally makes his or her own diagnosis and then starts treatment. Even when a patient is referred by someone else, the doctor will always take their own look. (…) I do not think there is any other field of care in which the health care provider takes care of the diagnostics and the physician then carries out the medical treatment. This requires trust in each other’s expertise (Individual interview).

## Discussion

This project described Dutch transgender care providers’ assessment of MCD sessions effectiveness in their clinical work with adolescents. A representative group of 34 team members from two teams in different cities participated in this mixed methods evaluation study, enabling us to acquire a broad, but nuanced understanding of their experiences with six MCD sessions.

Our results showed that the care providers considered MCD a useful method that has helped them deal with care situations where they were uncertain which step was morally right or where they could not agree on a what was the best possible care. In the individual interviews and focus groups, team members indicated that the need for thorough reflection on work challenges is particularly critical now because the current focus on efficiency has cut into contemplation time. The team members indicated a need to devote time, structure (in the shape of a facilitator and a conversation method), and dialogue (instead of a polemical debate) to a thorough reflection on difficult cases. Yet, not all team members valued the various aspects of MCD in the same way. They disagreed on how much time should be spent on an MCD session and how MCD sessions should be structured. Their criticisms of the MCD process focused on: the length of time dedicated to discussing individual cases, the need for more practical and concrete results after MCD sessions and the lack of follow-up and integration of MCD into regular work process. These results are in line with other studies on MCD, which show that the follow-up, organization, and implementation of CES can be challenging in clinical practice (Finder & Bliton, 2011; Hartman, Inguaggiato, Widdershoven, Wensing-Kruger, & Molewijk, 2019a; Hartman et al., 2019b; Hem et al., 2015; Weidema, van Dartel, & Molewijk, 2016).

Team members were critical about whether or not the MCD’s dialogue, including the specific conversation method used, was a determining factor leading to the perceived results. They wondered whether these same results could be achieved simply by taking the time to deliberate on a case. The data obtained in this study do not allow us to draw any conclusions about whether time or MCD as such was a more determinant factor. Nevertheless, we can say that MCD probably adds value in highlighting the moral aspects of a case. Such elements do not necessarily emerge in regular case discussions but result from the MCD method, structure, and facilitator-led form. As a result, MCD helps participants to increase their moral reflection skills (Dauwerse, 2013). Most regular case discussions take just as much time, but seldom get beyond the clinical aspect of the case and seldom take into account the values and norms behind the clinical reasoning. In addition, the structure and focus of MCD sessions allows focusing on dialogue rather than on debate and gives participants room to constructively discuss differences of opinion and to reflect on morally complex or problematic cases (Molewijk, van Zadelhoff, Lendemeijer, & Widdershoven, 2008b). This contrasts with most regular case discussions, in which participants typically try to convince others of their own opinion in a heated debate. Such a setting tempts participants to repeat the same argument time and time again. A recent Dutch study showed that MCD sessions offer more of the hallmarks of good moral deliberation than regular case discussions, as a greater proportion of the participants’ statements were categorized as examples of moral focus, variety of argumentation, and open interaction (de Snoo-Trimp, Kremer, Jellema, & Molewijk, 2020a).

The MCD sessions analyzed in this study led to changes for the transgender clinics at both the treatment plan and general policy levels. The sessions resulted in concrete changes of treatment plans and contributed to several adjustments of the general transgender care policy (Hartman et al., 2019a, b). For example, changes were made as to how strictly clinics apply the age criterion for starting puberty suppressing treatment. Dutch transgender clinics strictly maintained a minimum age of 12 years for eligibility to start this treatment. Partly due to the outcomes of several MCD sessions focusing on cases of young children and puberty suppressing treatment, the clinics now apply the minimum age criterion more flexibly. This result is in line with other studies which show that MCD promotes the development, improvement, and implementation of guidelines and policies (Molewijk et al., 2008b). It should be noted that describing other changes in clinical policy resulting from the MCD sessions analyzed in this study is beyond the scope of the current study. Nevertheless, for more information about the integration of CES into the daily work processes at a Dutch transgender clinic see Hartman et al. (2019a, b).

The individual interviews and focus groups in this study led to concrete recommendations on improving the use and implementation of MCD. The recommendations were: to ensure the lessons learned from MCD sessions are followed up; to boost the sense of ownership and responsibility regarding actions to be taken; and to ensure the lessons learned are reflected in guidelines and policies. To put these recommendations into action, in our local situation a steering group was created with members from the management team of the Center of Expertise for Gender Dysphoria at the Amsterdam University Medical Centers, location VUmc in Amsterdam. Two of the researchers discussed the recommendations with the steering group. Together, they decided to make adjustments to the MCD sessions themselves (e.g., focusing more on “who will do what” at the closure of each session) and to see to a deeper embedding of MCD in the work processes of the Center of Expertise for Gender Dysphoria at the Amsterdam University Medical Centers, location VUmc in Amsterdam (Hartman et al., 2019a, b).

The quantitative results from the Euro-MCD questionnaire, in which 26 possible outcomes of MCD are described (Svantesson et al., 2014), showed participants’ preferences for MCD outcomes and which MCD-related outcomes the participants actually observed during and after the MCD sessions. The results confirmed that the care providers expressed a particularly strong wish to see “mutual understanding,” “more open communication,” and a greater ability to “decide on concrete actions” as MCD outcomes. Most participants indeed noted an improvement in “mutual understanding” and “open communication” after participating in the MCD sessions. Furthermore, after participating in the MCD sessions, a majority of the team members experienced an “enhancement in mutual respect among the team members” and “became more aware of the stakeholders’ different perspectives” and of “recurring, ethically difficult situations.” Participants reported that spending more time reflecting on other team members’ thinking gave them a greater awareness of other stakeholders’ perspectives on the MCD case in question, which enhanced the trust within the team. These results are in line with other studies, which show that MCD can help teams more effectively deal with moral challenges and enhance collaboration and mutual trust (Hem et al., 2015; Janssens et al., 2014; Molewijk et al., 2008c; Weidema et al., 2015).

Besides the developments in the field of MCD, research also focuses on developing other new and innovative forms of CES. For example, forms of CES which can be used by sole practitioners. Such as ethics consultation (Aulisio, Arnold, & Youngner, 2000; Molewijk, Slowther, & Aulisio, 2016) and a moral compass which can be used individually (Hartman, Metselaar, Molewijk, Edelbroek, & Widdershoven, 2018).

Ever since the research for this study was conducted, CES has become a standard part of transgender care and MCD has been added to the CES toolbox. MCD is now part of policy days, in ad hoc situations, and occasionally in educational settings such as one-day professional courses on decision-making competence. In addition to MCD, several other methods have been added to the CES toolbox as well (Hartman et al., 2019a, b). Moreover, since this research was conducted one member of each team has been trained as an MCD facilitator, which enables the teams to use MCD in an ad hoc fashion whenever so desired. As such, this evaluation study enabled the professionals at the Amsterdam and Leiden teams to shape and embed CES as they themselves saw fit. This can be seen as part of an “integrative approach” to CES (Hartman et al., 2019a).

Till now, at the transgender clinic, it is not standard to involve patients in MCD sessions. However, participation of patients themselves in MCD sessions might bring to light new viewpoints on the moral issue at hand. The participation of transgender people’s own practical experiences and normative considerations can then be taken into account and might stimulate reflection and deepen decision-making processes even more. This could also enlarge the understanding of each others’ perspectives on good care. Therefore, participation of transgender people seen in the clinic and/or from the community at large in MCD sessions would be a great next step.

The current study showed that most team members at the transgender clinics in the Netherlands have positive experiences with MCD. Other studies show that MCD is also considered valuable by professionals in other branches of care, both in the Netherlands and internationally (de Snoo-Trimp et al., 2020b). However, we cannot assume that MCD is appreciated by transgender care providers outside the Netherlands. Therefore, we would encourage the collection of more qualitative and quantitative data on how transgender clinics in other countries experience CES and MCD. It would be worthwhile to explore what kind of support CES in general and MCD in particular can offer in other countries. A European study on the experiences of MCD and their outcomes highlights considerable differences in Europe, regarding the experiences with MCD outcomes and the rating of various MCD outcomes in terms of their importance to care providers (Svantesson et al., 2019). An international study focusing specifically on the relevance of CES in transgender care would show us whether there are cultural differences regarding: (1) the types of moral challenges transgender teams from other countries are confronted with; (2) how these challenges are framed; and (3) how the teams deal with these moral challenges.

### Strengths and Limitations

The present study had strengths and weaknesses. The mixed methods nature of this study enabled us to find out, in depth, how professionals who provide care for transgender adolescents in the Netherlands evaluated MCD. The qualitative data and quantitative data were mutually supportive. Furthermore, the diversity of the MCD participants enabled us to record a wide variety of care providers’ experiences and considerations. Nevertheless, the participants in this study were solely from transgender clinics in the Netherlands. Besides, only six members of the Amsterdam and Leiden teams were interviewed individually, in order to reduce the burden on the clinicians. Despite the small number of interviewees, we believe that due to our careful selection of participants our study results reflect the views of a representative group.

### Conclusion

In this mixed method study, Dutch transgender care providers evaluated the use of MCD as a form of ethics support. Although respondents were critical of the length of time spent on MCD, the lack of follow-up on insights gained from MCD sessions, and of the determining factors of the MCD sessions, they widely felt that it helped them to more effectively deal with moral challenges and that it contributed to improved mutual understanding, respect, and communication among their team members. Given the inherent ethical dimension of transgender care, especially in the care for children and adolescents in which the treatment can have lifelong consequences, and where treatment decisions are often surrounded by complex moral controversies and uncertainties, MCD appears to be a valuable addition to current treatment models in transgender care. MCD offers a trained facilitator, who is neutral, and a specific conversation method that make it easier and more profound to reflect upon the moral dimension of specific complex decisions. During MCD, the professionals’ reasoning and knowledge are included, yet MCD makes (possible conflicts of) underlying norms and values explicit and gives suggestions how to handle the uncertainty or disagreement within a team. As such, MCD can be seen as an additional tool that can be used in complex cases. For future research, it would be worthwhile to compare the usual decision-making process by transgender teams with decision-making processes that include the use of MCD more systematically. Finally, more research is needed which focuses on the actual contribution of MCD to the improvement of care quality (including its determining factors), the involvement of transgender people in MCD, and on how to integrate CES more into daily work processes.

## Appendix 1

List of steps in the dilemma method* (Amsterdam University Medical Centers, location VUmc in Amsterdam)

1. Moral case is presented
2. [Formulation of a general moral question]
3. Short formulation of the case presenter’s dilemma: Should I do A or B?
4. Opportunity for clarification & questions
5. Scheme with “perspectives,” “values” and “norms”
6. Brainstorm on possible alternatives
7. Round of individual answers to the dilemma question
8. Discuss possible group consensus, disagreement or decision (“weigh” values & norms)
9. Make practical appointments and plan date to evaluate these appointments
10. Evaluation of MCD content and process, also considering the facilitator’s role
  - *Molewijk, B. A. C., Abma, T. A., Stolper, M., & Widdershoven, G. A. M. (2008a). Teaching ethics in the clinic. *Journal of Medical Ethics, 34*, 120-124.
  - *Molewijk, B. A. C., & Ahlzen, R. (2011). Should the school doctor contact the mother of a 17-year-old girl who has expressed suicidal thoughts? *Clinical Ethics, 6*, 5–10.
  - *Stolper, M., Molewijk, B. A. C., & Widdershoven, G. A. M. (2016). Bioethics education in clinical settings: theory and practice of the dilemma method of moral case deliberation. *BMC Medical Ethics, 17*, 1–10.

## Appendix 2

Impression of the Euro-MCD questionnaire*

Instructions: Below is a list of possible outcomes from a Moral Case Deliberation session. Please indicate how important you consider each outcome in terms of how much it would strengthen you and your co-workers’ ability to manage ethically difficult situations. The list includes outcomes that may occur during MCD sessions and/or afterward in everyday clinical practice.

1. Develops my skills in analyzing ethically difficult situations
2. More open communication among co-workers
3. Co-workers reach a consensus on how to manage ethically difficult situations
4. Enables me to better manage the stress caused by ethically difficult situations
5. Contributes to the development of practice/policies in the workplace
6. Gives me more courage to express my ethical views
7. I feel more secure to express doubts or uncertainty regarding ethically difficult situations
8. Better mutual understanding of reasoning and behavior
9. I see ethically difficult situations from different perspectives
10. My co-workers and I become more aware of recurring, ethically difficult situations
11. Increases my awareness of the complexity of ethically difficult situations
12. Enhances my understanding of ethical theories (ethical principles, values and norms)
13. Enables me and my co-workers to decide on concrete steps to manage ethically difficult situations
14. Greater opportunity for everyone to have their say
15. Creates more opportunity to share difficult emotions and thoughts with co-workers
16. I can see more courses of action to manage ethically difficult situations
17. I listen more seriously to others’ opinions
18. Increases awareness of my own emotions regarding ethically difficult situations
19. Boosts my self-confidence when managing ethically difficult situations
20. Develops my ability to identify the core ethical question in difficult situations
21. My co-workers and I examine existing practice/policies in the workplace/organization more critically
22. My co-workers and I manage disagreements more constructively
23. I have a better understanding of my own responsibility in ethically difficult situations
24. Enhances mutual respect among co-workers
25. I become more aware of my preconceived notions
26. I understand better what it means to be a good professional
  - *Svantesson, M., Karlsson, J., Boitte, P., Schildmann, J., Dauwerse, L., Widdershoven, G. A. M., … Molewijk, B. A. C. (2014). Outcomes of Moral Case Deliberation. The development of an evaluation instrument for clinical ethics support (the Euro-MCD). *BMC Medical Ethics, 15*, 30.
